# Melatonin Attenuates Intervertebral Disk Degeneration *via* Maintaining Cartilaginous Endplate Integrity in Rats

**DOI:** 10.3389/fphys.2021.672572

**Published:** 2021-06-14

**Authors:** Xiexing Wu, Yijie Liu, Jiacheng Du, Xiaoping Li, Jiayi Lin, Li Ni, Pengfei Zhu, Hong Zhou, Fanchen Kong, Huilin Yang, Dechun Geng, Haiqing Mao

**Affiliations:** ^1^Department of Orthopedics, The First Affiliated Hospital of Soochow University, Suzhou, China; ^2^Department of Clinical Education, The First Affiliated Hospital of Soochow University, Suzhou, China; ^3^Department of Orthopedics Center, Ningbo No. 2 Hospital, Ningbo, China

**Keywords:** intervertebral disk degeneration, cartilaginous endplates, melatonin, osteoclastogenesis, inflammation, matrix degradation, NF-κB

## Abstract

**Objective:**

The aim of this study is to verify whether melatonin (Mel) could mitigate intervertebral disk degeneration (IVDD) in rats and to investigate the potential mechanism of it.

**Method:**

A rat acupuncture model of IVDD was established with intraperitoneal injection of Mel. The effect of Mel on IVDD was analyzed *via* radiologic and histological evaluations. The specific Mel receptors were investigated in both the nucleus pulposus (NP) and cartilaginous endplates (EPs). *In vitro*, EP cartilaginous cells (EPCs) were treated by different concentrations of Mel under lipopolysaccharide (LPS) and Luzindole conditions. In addition, LPS-induced inflammatory response and matrix degradation following nuclear factor kappa-B (NF-κB) pathway activation were investigated to confirm the potential mechanism of Mel on EPCs.

**Results:**

The percent disk height index (%DHI) and MRI signal decreased after initial puncture in the degeneration group compared with the control group, while Mel treatment protected disk height from decline and prevented the loss of water during the degeneration process. In the meantime, the histological staining of the Mel groups showed more integrity and well-ordered construction of the NP and EPs in both low and high concentration than that of the degeneration group. In addition, more deep-brown staining of type II collagen (Coll-II) was shown in the Mel groups compared with the degeneration group. Furthermore, in rat samples, immunohistochemical staining showed more positive cells of Mel receptors 1a and 1b in the EPs, instead of in the NP. Moreover, evident osteochondral lacuna formation was observed in rat EPs in the degeneration group; after Mel treatment, the osteochondral destruction alleviated accompanying fewer receptor activator for nuclear factor-κB ligand (RANKL) and tartrate-resistant acid phosphatase (TRAP)-stained positive cells expressed in the EPs. *In vitro*, Mel could promote the proliferation of EPCs, which protected EPCs from degeneration under LPS treatment. What is more, Mel downregulated the inflammatory response and matrix degradation of EPCs activated by NF-κB pathway through binding to its specific receptors.

**Conclusion:**

These results indicate that Mel protects the integrity of the EPs and attenuates IVDD by binding to the Mel receptors in the EPs. It may alleviate the inflammatory response and matrix degradation of EPCs activated by NF-κB pathway.

## Introduction

Low back pain (LBP) is a universal health problem. It is estimated that approximately 70% of adults will suffer from LBP at least once in their lifetime (Burton et al., 2006). LBP causes both tremendous psychological and financial burdens for patients, families, and society as a whole (Hong et al., 2013). The most common cause of LBP is intervertebral disk (IVD) degeneration (IVDD) (Luoma et al., 2000). The pathogenesis of IVDD is complicated and includes oxidative stress, inflammatory response, and apoptosis. Most studies focus on the pathological change of the nucleus pulposus (NP). However, few studies have examined the relationship between cartilaginous endplates (EPs) and IVDD. Although several methods, such as physical exercise, medical intervention, surgical treatment, and gene therapy are used to treat IVDD, none of them can effectively restore degenerated disks (Sakai et al., 2005).

Anatomically, an IVD consists of the NP, annulus fibrosus (AF), and EPs. The NP is mainly composed of two types of cells, termed notochordal cells and chondrocyte-like cells (Colombier et al., 2014). The former can promote the proliferation of the latter and induce the synthesis of extracellular matrix (ECM), which is rich in type II collagen (Col-II) and proteoglycans. The AF is a structure that encapsulates the NP and provides mechanical support for the integrity of the NP. The AF is mainly composed of water, types I and II collagen fibers, proteoglycans, and proteins. The upper and lower EPs are composed of hyaline cartilage and are located at the adjacent vertebral edge. It distributes disk pressure to adjacent vertebrae, which plays a critical role in dispersing mechanical stress. In addition, the EPs are permeable, which is vital for exchange of glucose, oxygen, and metabolites in the IVD (Fontana et al., 2015). DeLucca et al. (2016) found that EP degeneration leads to a 50%–60% decrease in EP permeability. Therefore, the physiological functions of the NP, AF, and EPs are indispensable for maintaining the morphological and functional integrity of the IVD.

Melatonin (*N*-acetyl-5-methoxytryptamine; Mel) is a major neuroendocrine hormone secreted by the vertebrate pineal gland, which plays a fundamental role in many processes in the human body (Reiter, 1991; Reiter et al., 1995). Mel takes effect by binding to its corresponding Mel receptors, which are divided into types 1a and 1b. Type 1a receptors are mainly expressed in mammals, while type 1b Mel receptors are expressed to a lesser extent (Brzezinski, 1997). Known physiological effects of Mel include improving sleep quality, regulating heart rate, and maintaining body temperature (Maestroni, 1993; Pandi-Perumal et al., 2008). It has also been reported that Mel plays a key role in osteoarthritis *via* regulating bone formation, mineralization, and remodeling (Histing et al., 2012; Hong et al., 2014). Mel has been shown to have anti-inflammatory, anti-oxidative, and anti-apoptotic effects in animal models of osteoarthritis (Reiter et al., 1995; Mazzon et al., 2006; Chen et al., 2016). In addition to regulating bone metabolism, Mel also shows a potential protective effect on articular chondrocytes (Zhang et al., 2014), which have a composition and function similar to those of EP chondrocytes (EPCs) (Muir, 1995). As mentioned previously, the chondrocyte-like cells in the EPs have a composition and function similar to those of the articular chondrocytes (Colombier et al., 2014). Therefore, we supposed that Mel may be protective against IVDD.

In this study, it was confirmed that Mel delayed IVDD in a rat caudal acupuncture model, *via* radiologic and histological analyses. In addition, immunohistochemical staining indicated that Mel receptors were predominantly expressed in the EPs rather than in the NP in rats. Tartrate-resistant acid phosphatase (TRAP) staining showed osteoclast recruitment mainly in the EPs, and the number of TRAP-positive cells significantly decreased following treatment with high and low concentrations of Mel compared with that of the degeneration group. It indicated that Mel delayed the IVD degeneration through binding to Mel receptors in the EPs as well as inhibiting osteoclastogenesis. The *in vitro* studies showed that Mel alleviated the inflammatory response and matrix degradation of EPCs activated by nuclear factor kappa-B (NF-κB) pathway through binding to its specific receptors.

## Materials and Methods

### Endplate Cartilage Culture

Male Sprague-Dawley rats (6 weeks) were purchased from the Animal Experimental Center of Soochow University. After euthanasia with excessive pentobarbital (0.5 g/kg), the caudal vertebrae of the rats were collected. After the NP and AF tissues were removed, the EP cartilage tissue was exposed and collected. The tissues were digested by 0.5% type II collagenase overnight in 37°C incubator. After centrifugation at the second day, the precipitates were resuspended with Dulbecco’s modified Eagle medium/nutrient mixture F-12 (DMEM/F-12; Invitrogen, Waltham, MA, United States), with 15% fetal bovine serum (FBS; Invitrogen) and 1% penicillin/streptomycin, and then seeded in six-well plates. The cells were cultured in an incubator containing 5% carbon dioxide at 37°C. After fusion, the cells were digested with 0.25% trypsin EDTA (Invitrogen) and planted in a 10-cm-diameter culture dish.

### RT-qPCR

TRIzol reagent (Invitrogen) was used to extract total RNA from different groups of EPCs, and NanoDrop 2000 (Thermo Fisher Scientific, Waltham, MA, United States) was used to quantify the total RNA concentration. Then, we used PrimeScript RT Master Mix Kit (Takara, Kusatsu, Japan) to carry out the inversion rate, so that the total amount of reverse transcripts (cDNA) reached 1 μg/20 μl and amplified with this product. The PCR amplification system was 20 μl, including 10 μl of major mixture of Forget-Me-Not qPCR (Biotium, Inc., Hayward, CA, United States), 0.5 μl of forward and reverse primers, 1 μl of cDNA, and 8 μl of RNase-free distilled water (ddH_2_O; Invitrogen). Cfx96 touch real-time PCR detection system (Bio-Rad Laboratories, Hercules, CA, United States) was used. The circulation threshold was normalized to glyceraldehyde 3-phosphate dehydrogenase (GAPDH) level. To calculate the mRNA level, we used the 2^−ΔCT^ method. Primer sequences are shown in Table 1.

**TABLE 1:** Primers used for RT-PCR.

Gene	Primer/probe	Sequence
Col-II	Forward	GCTCATCCAGGGCTCCAATG
	Reverse	CAATGGGAAGGCGTGAGGTC
MMP13	Forward	TCCATCCCGAGACCTCATGT
	Reverse	AGCATCATCATAACTCCACACG
IL-1β	Forward	GACTTCACCATGGAACCCGT
	Reverse	GGAGACTGCCCATTCTCGAC
MMP3	Forward	AAAGAACCCGCTGAGAGCAG
	Reverse	AACCTCCATGCCAGCATCTT
ADAMTS-5	Forward	GCCTGCAAGGGAAATGTGTG
	Reverse	GGCGGAAAGATTTGCCGTTAG
GAPDH	Forward	GACATGCCGCCTGGAGAAAC
	Reverse	AGCCCAGGATGCCCTTTAGT

### Cell Viability Assay

In order to evaluate the effect of lipopolysaccharide (LPS), Mel, and Luzindole (a non-selective Mel receptor inhibitor) on the proliferation of EPCs, we used cell counting kit-8 (CCK-8; Dojindo Co., Kumamoto, Japan). The EPCs were seeded in 96-well plates at the density of 3 × 10^3^/well and then intervened with different concentrations of Mel for 1, 3, and 5 days. The value-added activity was detected by CCK-8 kit according to the instructions. Optical density (OD) was then measured at 450-nm wavelength. CCK-8 assay was also performed with different concentrations of Mel treatment under LPS and Luzindole condition after EPC culturing for 3 days.

### Western Blotting Assay

Radioimmunoprecipitation assay (RIPA) buffer and benzenesulfonyl fluoride (PMSF) (Beyotime, Haimen, Jiangsu, China) were used to extract the total protein from EPCs. After complete lysis, the solution was centrifuged, and the supernatant was taken out. The protein concentration was determined by bicinchoninic acid (BCA) kit. Then, the protein of different molecular weight was separated by 12 alkyl sulfate polyacrylamide gel electrophoresis, and the protein was transferred to Bio-Rad Laboratories (Hercules, CA, United States). After being blocked with QuickBlock^TM^ Blocking Buffer (Beyotime), the different bands were incubated overnight in the following primary antibodies: anti-β-actin (1:5,000, af5001, Beyotime), anti-Col-II (1:1,000, ab34712, Abcam, Cambridge, United Kingdom), anti-matrix metalloproteinase-13 (anti-MMP13) (1:3,000, ab39012, Abcam), anti-interleukin-1β (anti-IL-1β) (1:2,000, ab205924, Abcam), anti-matrix metalloproteinase-3 (anti-MMP3) (1:5,000, ab52915, Abcam), anti-a disintegrin-like and metalloproteinase with thrombospondin motifs-5 (ADAMTS-5) (1:250, ab41037, Abcam), anti-p-P65 (1:2,000, ab194726, Abcam), and anti-P65 (1:1,000, ab16502, Abcam). At the second day, the membrane was washed with TBST (Beyotime) to remove the unbound primary antibody, and then the corresponding secondary antibodies (a0288 and a0279, Beyotime) were added and cultured for 1 h. Finally, the band was detected by Thermo Fisher Scientific.

### Immunocytochemistry Staining

EPCs were seeded into 24-well plates (climbing piece was put in advance). After treatment, 4% paraformaldehyde was added and fixed at 4°C for 20 min. Then, Triton X-100 (Beyotime) was added and incubated at room temperature for 10 min. Subsequently, 500 μl of blocking solution (quick blocking buffer, Beyotime) was added to each well for blocking at room temperature. After being blocked, the following primary antibodies were added to each well: anti-Col-II (1:1,000, ab34712, Abcam), anti-MMP13 (1:2,000, ab219620, Abcam), and anti-P65 (1:200, ab16502, Abcam). The 24-well plates were placed at 4°C overnight. At the second day, the primary antibody was recovered, an appropriate amount of corresponding fluorescent secondary antibody (either Alexa Fluor^®^ 488 or Alexa Fluor^®^ 647, Abcam) was added, and the samples were cultured at 37°C for 1 h. Then, DAPI was added for nucleus labeling. Finally, the cell slide was placed on the glass slide, sealed with anti-fluorescence quenching agent, and observed under high-resolution microscope.

### Ethics Statement

A total of 80 male Sprague-Dawley rats (3 months, 450 ± 50 g) were acquired from the Soochow University Animal Center. Rats were housed in a 12-h light/dark cycle, and room temperature was maintained at 21°C, with food and water provided *ad libitum*. The animal study was reviewed and approved by the Ethics Committee of the First Affiliated Hospital of Soochow University (No. 201801A005). Human IVD tissues were obtained from the lumbar disks of scoliosis patients undergoing deformity correction surgery. The studies involving human participants were reviewed and approved by the Ethics Committee of the First Affiliated Hospital of Soochow University (No. 2019254). The patients/participants provided their written informed consent to participate in this study.

### Operation Procedures and Groups

Twenty rats were randomly assigned to the control group. Sixty rats were assigned to study groups. These rats were anesthetized with pentobarbital administered intramuscularly (0.1 g/kg) after 12 h of fasting and 4 h of water deprivation. A 20-gauge (20-G) needle was used to puncture each rat between the 7th and 8th caudal vertebrae (Co7–8), the 8th and 9th caudal vertebrae (Co8–9), and the 9th and 10th caudal vertebrae (Co9–10) (Han et al., 2008). As described in a previous study, the needle pierced two layers of AF, rotating for 5 s, and then held still for 30 s in order to achieve a good degenerative effect (Wu et al., 2018). The 60 rats were then randomly divided into a high-concentration Mel treatment group (50 mg/kg, *n* = 20), a low-concentration Mel treatment group (5 mg/kg, *n* = 20), and a phosphate-buffered saline (PBS) group (degeneration group, *n* = 20). The Mel concentrations chosen have both been shown to effectively inhibit osteoclastogenesis and activation induced by the receptor activator for nuclear factor-κB ligand (RANKL) in studies with mice (Koyama et al., 2002). Intraperitoneal injections of high- and low-dose Mel and PBS were administered between 16:00 and 17:00 once a day for 7, 14, 21, and 28 days.

### Radiography and MRI Scan

Five rats were randomly selected from each group to undergo X-ray and MRI examinations at 7, 14, 21, and 28 days after the puncture injury. Each rat was held in a horizontal, supine position, with a straightened tail, on a mammographic radiographic unit (GE Healthcare, Chicago, IL, United States). X-rays were performed with the following conditions: collimator-to-film 66 cm, penetration 35 kV, and exposure 63 mA. Cross-sectional T2-weighted MRI images were gathered *via* a 1.5-T system (GE; fill time 3,000 ms; echo time 80 ms; field of view 200 mm × 200 mm; and scan thickness 1.4 mm).

### Radiographic Assessments and MRI Analysis

A medical imaging tool (DICOM3.0, Neusoft^®^ PACS/RIS, China) was utilized to process the radiologic data. The procedure was completed by an experienced physician who was unaware of this study. Data on IVD height and adjacent vertebrae height were obtained *via* the above software. The disk height index (DHI) and percent DHI (%DHI) were then calculated (Masuda et al., 2005; Han et al., 2008). With the improved Thompson classification applied, MRI images were classified from I to IV (I, normal; II, signal intensity decreased slightly with high signal regions narrowed significantly; III, signal intensity decreased moderately; and IV, signal intensity decreased severely) *via* evaluating the signal intensity of the T2-weighted images.

### Micro-CT Imaging

After radiographic and MRI scans at 7, 14, 21, and 28 days after the puncture injury, rats were euthanized *via* intraperitoneal injection with an excess pentobarbital intramuscularly administered (0.5 g/kg). Rat caudal specimens were then obtained using a scalpel blade and fixed in a neutral 10% formalin solution for 48 h and then subjected to micro-computed tomography (micro-CT) scanning (*n* = 5 per group) using a high-resolution micro-CT from SkyScan (SkyScan 1176, Aartselaar, Belgium). The program was set to a scanning thickness of 9 μm at a voltage of 50 kV with a current of 500 μA and a regular increment of 0.7° in 180 rotation steps. Each scan took approximately 13 min. A 3D reconstruction was performed using a CT analyzer (SkyScan), and various histomorphometric measurements were calculated as follows: bone mineral density (BMD, mg/cm^3^) and ratio of bone volume to tissue volume (BV/TV%).

### Histological and Immunohistochemical Staining

Following euthanasia, all IVDs were obtained from each rat, fixed in 4% formaldehyde solution for 48 h, decalcified by 10% ethylenediaminetetraacetic acid (EDTA, Sigma) for 40 days, and finally embedded in paraffin. The paraffin-embedded IVD specimens were cut into 5-μm coronal sections with intact structures. Parts of these specimens were stained with safranin-O fast green and hematoxylin and eosin (H&E). The method established by Masuda was used to assess the histological morphology of the specimens (Table 2; Masuda et al., 2005).

**TABLE 2:** Definition of histological scale.

**I. Anulus fibrosus**
**Grade:**
1. Normal, pattern of fibrocartilage lamellae (U-shaped in the posterior aspect and slightly convex in the anterior aspect) without ruptured fibers and without a serpentine appearance anywhere within the anulus 2. Ruptured or serpentined patterned fibers in less than 30% of the anulus 3. Ruptured or serpentined patterned fibers in more than 30% of the anulus
**II. Border between the anulus fibrosus and nucleus pulposus**
**Grade:**
1. Normal 2. Minimally interrupted 3. Moderate/severe interruption
**III. Cellularity of the nucleus pulposus**
**Grade:**
1. Normal cellularity with large vacuoles in the gelatinous structure of the matrix 2. Slight decrease in the number of cells and fewer vacuoles 3. Moderate/severe decrease (> 50%) in the number of cells and no vacuoles
**IV. Matrix of the nucleus pulposus**
**Grade:**
1. Normal gelatinous appearance 2. Slight condensation of the extracellular matrix 3. Moderate/severe condensation of the extracellular matrix

TRAP staining was performed with a TRAP staining kit (#387A, Sigma-Aldrich, St. Louis, MO, United States). Dark purple particles were considered osteoclasts. A new method by Sawyer was used to quantify TRAP-positive cells (Sawyer et al., 2003). Immunohistochemical staining was used to observe specific expression of anti-Col-II (1:500, ab34712, Abcam), MMP13 (1:200, ab219620, Abcam), IL-1β (1:500, ab205924, Abcam), anti-p-P65 (1:200, ab16502, Abcam), and RANKL (1:500, ab239607, Abcam) in the rat IVD tissues. After being dewaxed, gradient hydrating and protease-modifying, sections were blocked with 5% horse serum and then incubated with the primary antibody overnight at 4°C. Sections were then washed with PBS, then successively incubated with secondary antibody and tertiary antibodies for 30 min, stained with 3,3′-diaminobenzidine tetrahydrochloride, and finally counterstained with hematoxylin. All images were taken using a high-resolution microscope. The positive staining cells and areas were analyzed using ImageJ software.

### Statistical Analysis

All data were calculated using SPSS 17.0, and the results are expressed as mean ± standard deviation. Differences between two groups were compared using two-tailed Student’s *t*-test, and differences among groups were compared using one-way analysis of variance (ANOVA). The Mann–Whitney U-test and the Kruskal–Wallis tests were used to determine the impacts of treatment and time on non-parametric data, such as histological and MRI grade, for each parameter. Values were considered statistically significant at P level < 0.05.

## Results

### Melatonin Ameliorated Both Nucleus Pulposus and Endplate Degeneration in Rats

**FIGURE 1:**
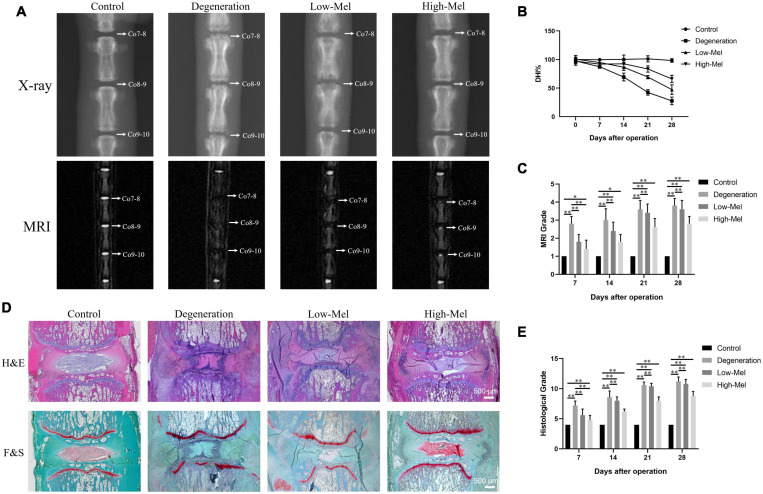
Radiographs and histological analysis of intervertebral disks (IVDs) in the rat intervertebral disk degeneration (IVDD) model. **(A)** X-ray photographs and MRI scans of IVDs on the 7th day after puncture injury. **(B)** The change in %DHI over time among groups. **(C)** MRI grading for all groups over time. **(D)** H&E staining and safranin-O staining. **(E)** Histological score on the 7th, 14th, 21st, and 28th days after puncture injury (*n* = 5, ^∗^*P* < 0.05, ^∗∗^*P* < 0.01).

The effect of Mel was initially assessed by imageological examination. X-ray examinations and MRI were performed at 7, 14, 21, and 28 days after puncture. The disk height of rats that underwent puncture began to decline at the seventh day after puncture injury, whereas disk height in rats in the control group remained unchanged. As shown in Figure 1A, the degeneration group exhibited apparent narrowing in the pierced IVD level, and the EPs became rough and rugged with bone destruction compared with the control group. Rats in the groups treated with Mel, especially rats in the high concentration group, maintained disk height and EP integrity. The %DHI at 7 days after puncture only decreased to 86.4% ± 5.4% and 92.6% ± 5.5% in the low- and high-Mel concentration groups compared with the control group, while 69.4% ± 6.8% decline was shown in the degeneration group (Figure 1B). The MRI signal in the degeneration group was significantly lower than in the control group, indicating that water content in the disks was missing in this group. Rats in the Mel treatment groups exhibited stronger MRI signals than did the degeneration group. Semi-quantification by MRI grade showed that MRI scores in both the low- and high-concentration Mel groups were significantly lower than in the degeneration group at 7 days (Figure 1C). These results indicated that Mel treatment alleviated puncture-induced IVDD in rats.

Histological staining was performed to explore further remarkable effects of Mel during disk degeneration. As shown in Figure 1D, 7 days after modeling, the needle puncture caused a loss of the NP; therefore, the volume of the NP reduced and the AF lamellar arrangement became distorted and warped in the degeneration group. Furthermore, the EPs became calcified, and ossification developed. The capillaries supplying nutrition fused and disappeared in the degeneration group. After Mel treatment, especially in the high concentration group, relatively complete NP tissues were preserved with unbroken AF and EPs. Safranin-O staining indicated that vacuolar cells in the NP have fibrocartilaginous changes after needle puncture. Furthermore, the wavy basophilic tidemark of the growth plate became interrupted. Mel treatment prevented the loss of collagen and proteoglycan in both the NP and EPs, thus alleviating their degeneration process. The histological scores in the low- and high-Mel groups were significantly lower than in the degeneration group (Figure 1E).

**FIGURE 2:**
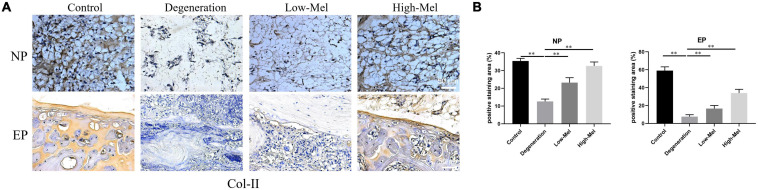
Melatonin (Mel) treatment reduced the degradation of extracellular matrix (ECM). **(A)** Immunohistochemical staining of type II collagen (Col-II) in both nucleus pulposus (NP) tissues and endplate (EP) tissues of rats on the 7th day after puncture injury. **(B)** Semi-quantitative analysis of Col-II on the 7th day after puncture injury (*n* = 5, ^∗^*P* < 0.05, ^∗∗^*P* < 0.01).

Immunohistochemical staining of Col-II on the NP and EPs was performed to evaluate the degree of IVDD in different groups. In both NP and EP areas, abundant deep brown DAB staining of the Col-II area was interlaced and arranged in the control and high-concentration Mel groups. Conversely, the low-concentration Mel and degeneration groups had fewer positively stained regions, indicating degradation and remodeling of the ECM. The amounts of Col-II-positive areas in the low- and high-Mel groups were 1.8- and 2.6-fold in the NP, 2.2- and 4.5-fold in the EPs, respectively, compared with the degeneration group (Figure 2). All the above data demonstrated the therapeutic effect of Mel for disk degeneration *in vivo*.

### Melatonin Attenuates Cartilage Destruction and Subchondral Bone Loss

**FIGURE 3:**
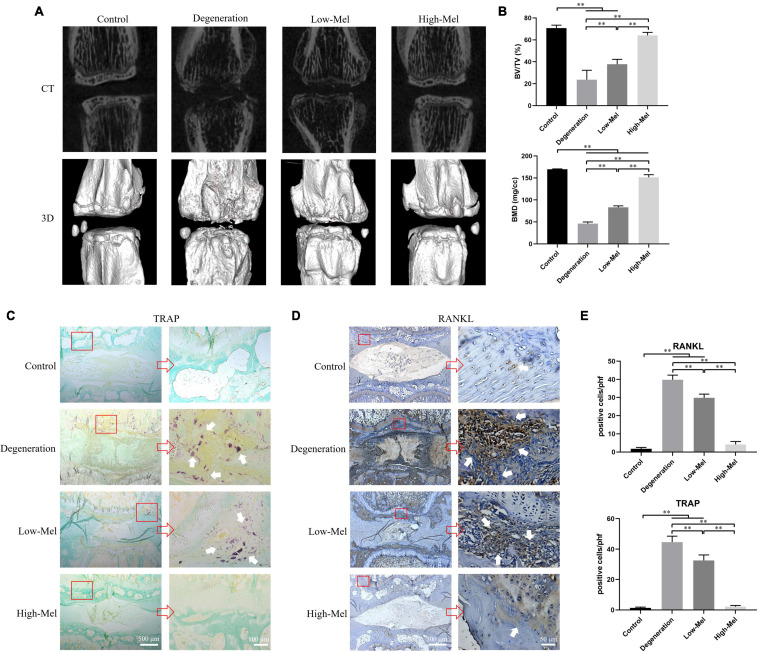
Melatonin (Mel) inhibited the destruction of endplate (EP) and reduced subchondral bone loss. **(A)** Micro-CT images and 3D surface reconstruction of rat caudal vertebrae among different groups on the 7th day. **(B)** Micro-CT analysis of specific indicators: bone volume to tissue volume (BV/TV) and bone mineral density (BMD). **(C)** Tartrate-resistant acid phosphatase (TRAP) staining of rat coccygeal intervertebral disk (IVD). **(D)** Immunohistochemical staining of receptor activator for nuclear factor-κB ligand (RANKL) in rat coccygeal IVD. **(E)** Quantitation of TRAP-positive cells and RANKL-positive cells among different groups (*n* = 5, ^∗^*P* < 0.05, ^∗∗^*P* < 0.01).

The EPs with structural integrity are the major nutrient source for IVDs (Fontana et al., 2015). Micro-CT 3D-reconstructed images showed that evident subchondral bone lacunae had formed in the EPs in the rats with puncture-induced disk degeneration. As expected, Mel treatment effectively attenuated subchondral bone defects in IVDD in a dose-dependent manner. Subchondral bone loss and destruction in the degeneration group are shown by coronal CT scans even more clearly; the loss and destruction were effectively mitigated by Mel treatment as well (Figure 3A). Quantitative analysis in Figure 3B showed a decrease of 66.5% in BV/TV and 72.8% in BMD in the degeneration group compared with the control group. When 5 or 50 mg/kg of Mel was daily injected, the BV/TV increased 1.6-fold or 2.7-fold and BMD increased 1.8-fold or 3.3-fold, respectively, compared with those in the degeneration group. Collectively, these findings suggest that Mel may exert an inhibitory effect on cartilage destruction and subchondral bone loss in the EPs during the progression of IVDD.

Osteoclasts, the core of bone resorption and destruction, initiate differentiated by RANKL (Goel and Vohora, 2020). TRAP staining revealed that plenty of mature osteoclasts were expressed in the EPs instead of the NP in rats that underwent needle puncture (Figure 3C). Mel treatment groups showed a significant and dose-dependent inhibition on dark-purple-stained osteoclasts as compared with those of the degeneration group. The number of TRAP positive cells was 26.9 and 95.1% lower, respectively, in the low- and high-concentration Mel groups compared with the degeneration group (Figure 3E). Consistent with TRAP staining, clear RANKL staining was found in the same regions as osteoclast expression in the degeneration group; it was also significantly decreased in the Mel groups (Figure 3D). Histomorphometric results showed that the number of RANKL-positive cells was 29.8 ± 2.1 and 4.2 ± 1.6 in the low- and high-Mel groups, respectively, compared with 39.8 ± 2.6 in the degeneration group (Figure 3E), demonstrating that Mel injection can inhibit RANKL expression and osteoclastogenesis in IVDD rats *in vivo*.

### Melatonin Targets Endplate Cartilage Through Melatonin-Specific Receptor in Rats

**FIGURE 4:**
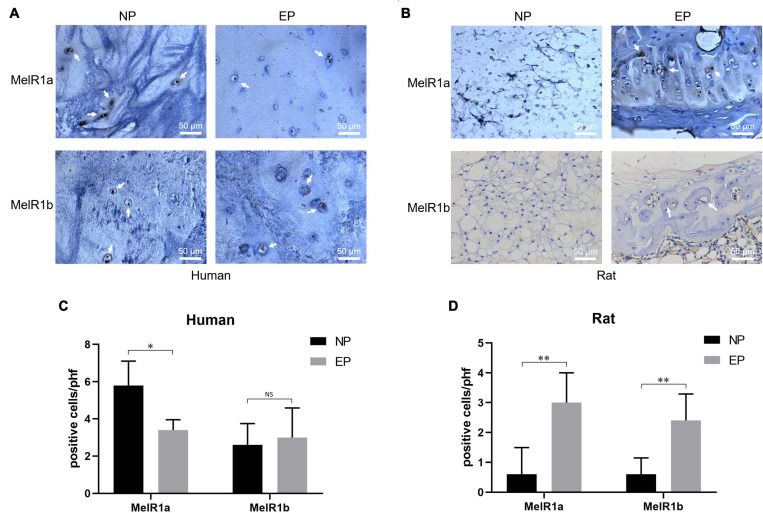
Immunohistochemical staining of melatonin (Mel) receptors 1a and 1b. **(A)** Mel receptors 1a and 1b were both expressed on nucleus pulposus (NP) and endplate (EP) of humans. **(B)** Mel receptors 1a and 1b were only expressed on EP rather than NP of rats. **(C)** Quantitation of Mel receptors 1a and 1b-positive cells in humans. **(D)** Quantitation of Mel receptors 1a and 1b-positive cells in rats [*n* = 5, no significant difference (NS), ^∗^*P* < 0.05, ^∗∗^*P* < 0.01].

Mel works by binding to the Mel receptor, which is extensively expressed in mammals (Witt-Enderby et al., 2006). Thus, to investigate the specific site where Mel acts to attenuate IVDD, the expression of Mel receptors 1a and 1b was identified in the region of the NP and EP in both rats and humans. As shown in Figure 4, Mel receptors 1a and 1b were expressed in both the human NP and EPs, while the expression of Mel receptor 1a in the NP was significantly higher than that in the EPs. However, the expression of Mel receptors 1a and 1b in rat NP tissue and EP tissue was completely opposite to that in humans, which may be due to species specificity. Both receptors were only observed in EP tissue in rats. Therefore, we could preliminarily speculate that Mel might take effect in alleviating IVDD in rats by targeting EP tissue instead of NP tissue. And further *in vitro* experiments were focused on the EPCs of rats.

### Melatonin Delays the Degeneration of Endplate Chondrocytes *via* Melatonin Receptors

**FIGURE 5:**
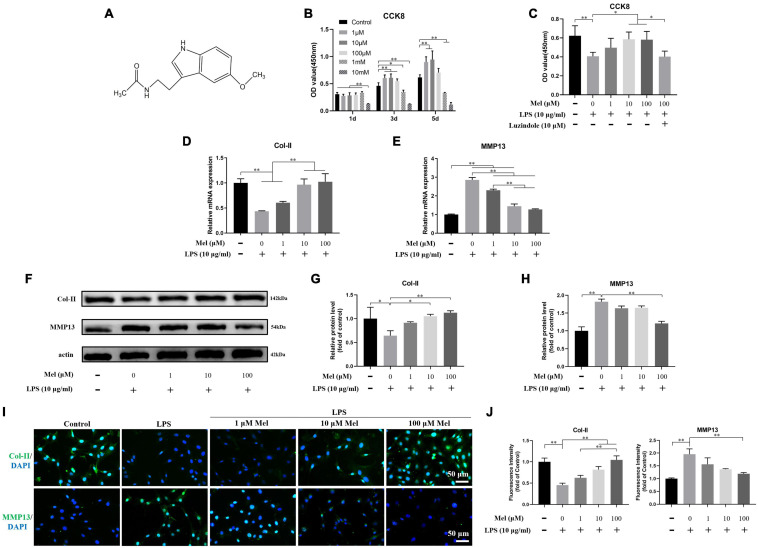
Melatonin (Mel) alleviated the degeneration of endplate cartilaginous cells (EPCs). **(A)** Molecular formula of Mel. **(B)** Optical density (OD) values (450 nm) of cell counting kit-8 (CCK-8) assay on EPCs under 1 μM, 10 μM, 100 μM, 1 mM, and 10 mM of Mel treatment at 1, 3, and 5 days. **(C)** OD values (450 nm) of CCK-8 assay on EPCs under lipopolysaccharide (LPS), Mel, and Luzindole treatment at 3 days. **(D,E)** qRT-PCR analysis of the relative mRNA expression of type II collagen (Col-II) **(D)** and matrix metalloproteinase-13 (MMP13) **(E)** under LPS and Mel treatment. **(F)** Western blotting for proteins of Col-II and MMP13 under LPS and Mel treatment. **(G,H)** Quantitation of relative protein level of Col-II and MMP13. **(I)** Immunofluorescence staining of Col-II and MMP13 under LPS and Mel treatment. **(J)** Quantitation of fluorescence intensity of Col-II and MMP13 (^∗^*P* < 0.05, ^∗∗^*P* < 0.01).

Mel belongs to indole heterocyclic compounds (Figure 5A). At low concentration (1, 10, and 100 μM), it had no obvious toxic effect on EPCs. On the contrary, it could stimulate the proliferation of EPCs. While the high concentration (1 and 10 mM) of Mel took a significant effect in the inhibition of cell proliferation, it became more obvious along with the passage of time (Figure 5B). We chose LPS to induce degeneration of EPCs by activating inflammatory response. The proliferation of EPCs was significant decreased following LPS treatment; as consistent with the results above, support by Mel reversed the cellular toxicity of LPS on EPCs, which was restricted by the non-selective receptor inhibition—Luzindole (Figure 5C).

The PCR and Western blotting (WB) results showed that LPS significantly induced the degeneration of EPCs, which was manifested in the decreased expression of Col-II and the increased expression of MMP13 (Figures 5D–H). However, Mel can significantly reverse the negative effects induced by LPS. ICC results further confirmed the role of Mel in the degeneration of EPCs. In the LPS group, the expression of Col-II was significantly decreased while the expression of MMP13 was significantly increased, which was reversed by Mel (Figures 5I,J). These results suggested that Mel could attenuate the degeneration of EPCs.

**FIGURE 6:**
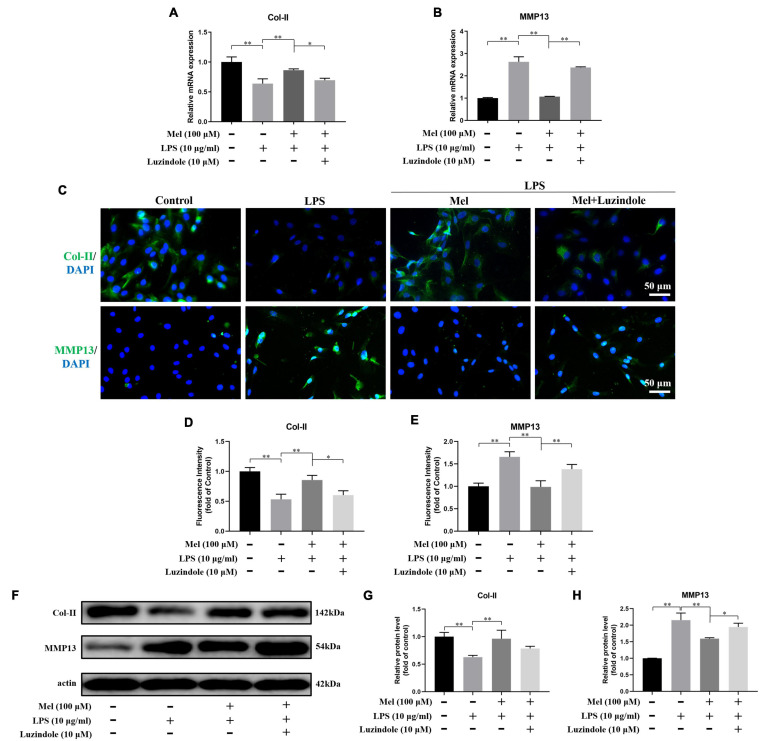
Melatonin (Mel) alleviated the degeneration of endplate cartilaginous cells (EPCs) through binding to its specific receptors. **(A,B)** qRT-PCR analysis of the relative mRNA expression of type II collagen (Col-II) **(A)** and matrix metalloproteinase-13 (MMP13) **(B)** under lipopolysaccharide (LPS), Mel, and Luzindole treatment. **(C)** Immunofluorescence staining of Col-II and MMP13 under LPS, Mel, and Luzindole treatment. **(D,E)** Quantitation of fluorescence intensity of Col-II and MMP13. **(F)** Western blotting for proteins of Col-II and MMP13 under LPS, Mel, and Luzindole treatment. **(G,H)** Quantitation of relative protein level of Col-II and MMP13 (^∗^*P* < 0.05, ^∗∗^*P* < 0.01).

Next, we tried to explore whether Mel affected the degeneration of EPCs by targeting Mel receptor. As shown in Figure 6, the positive effect of Mel on the degeneration of EPCs was significantly reversed after adding Luzindole. Specifically, compared with the Mel + LPS group, the relative mRNA expression of Col-II was significantly decreased following adding Luzindole, and the MMP13 increased correspondingly. Similarly, the fluorescence staining and WB also showed the blocking effect of Luzindole. Together, these results indicated that Mel took effect in inhibiting EPC degeneration through binding to its specific receptors.

### Melatonin Affects Inflammation and Matrix Degradation of Endplate Chondrocytes Through Nuclear Factor Kappa-B Pathway

**FIGURE 7:**
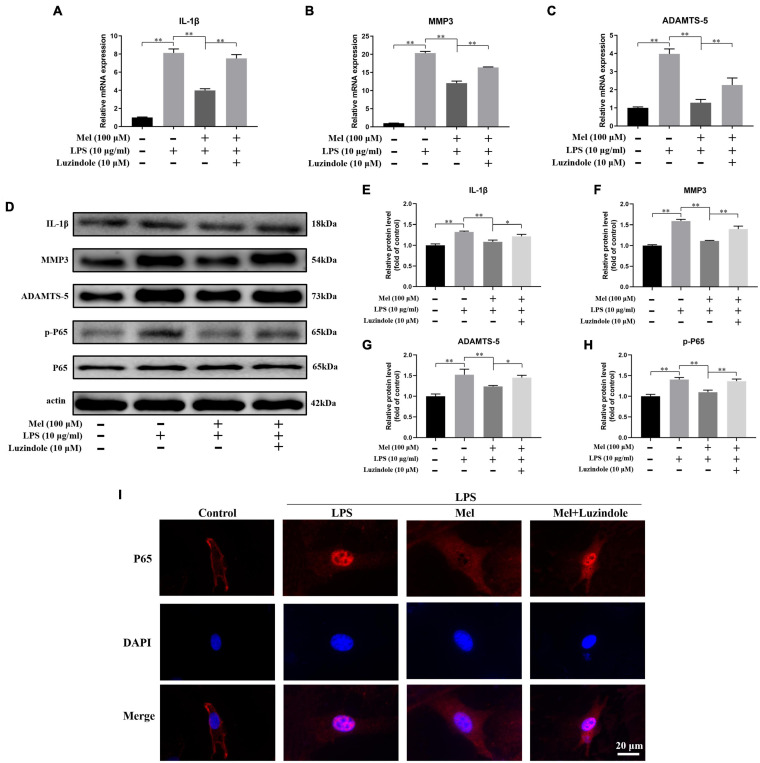
Melatonin (Mel) alleviated the degeneration of endplate cartilaginous cells (EPCs) through inhibiting inflammatory response and matrix degradation activated by NF-κB pathway. **(A–C)** qRT-PCR analysis of the relative mRNA expression of IL-1β **(A)**, matrix metalloproteinase-3 (MMP3) **(B)**, and ADAMTS-5 **(C)** under lipopolysaccharide (LPS), Mel, and Luzindole treatment. **(D)** Western blotting for proteins of IL-1β, MMP3, ADAMTS-5, p-P65, and P65 under LPS, Mel, and Luzindole treatment. **(E–H)** Quantitation of relative protein level of IL-1β, MMP3, ADAMTS-5, and p-P65. **(I)** Immunofluorescence staining of P65 nuclear translocation under LPS, Mel, and Luzindole treatment (^∗^*P* < 0.05, ^∗∗^*P* < 0.01).

In order to verify the effect of Mel on inflammation and matrix degradation of EPCs, we detected the changes of related markers. It could be seen from Figures 7A–G that LPS could significantly promote the expression of inflammatory cytokines (IL-1β) and ECM degradation-related genes (MMP3 and ADAMTS-5), while the addition of Mel significantly reversed this negative effect. Furthermore, Luzindole showed aggravation in the inflammation and ECM degradation of EPCs under Mel treatment.

NF-κB pathway is an important pathway in inflammatory response, and it was reported that this pathway is involved in the process of LPS-induced degeneration (Sadowska et al., 2018). However, whether Mel participates in the degeneration of EPCs activated by NF-κB signals is still unclear. Our results showed that the expression of p-P65 in the LPS group was significantly higher than that in control group, and Mel could significantly inhibit the phosphorylation of P65, which was reversed by Luzindole (Figures 7D,H). In addition, ICC results showed that P65 incorporation into nucleus of EPCs caused by LPS was significantly inhibited by Mel. However, Luzindole could reverse the inhibitory effect of Mel on P65 (Figure 7I). This suggests that NF-κB pathway is involved in the mechanism of Mel.

**FIGURE 8:**
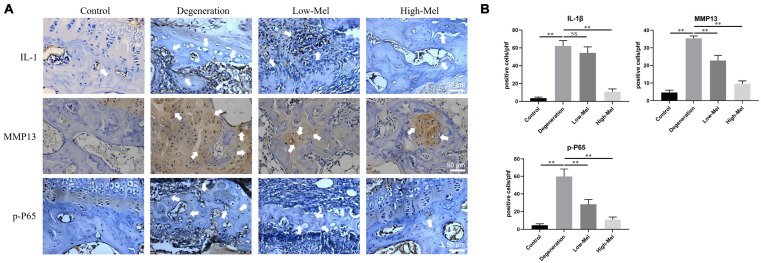
*In vivo* studies of melatonin (Mel) take effect on inflammatory response and matrix degradation activated by NF-κB pathway. **(A)** Immunohistochemical staining of IL-1β, matrix metalloproteinase-3 (MMP3), and p-P65 in rat endplate (EP) tissues. **(B)** Quantitation of IL-1β, MMP3, and p-P65 (*n* = 5, ^∗^*P* < 0.05, ^∗∗^*P* < 0.01).

Subsequently, we sought further verification in rat samples. The *in vivo* results were consistent with our *in vitro* results. Mel could inhibit the inflammatory response and the degradation of ECM of the EPs through NF-κB pathway, which exhibited the reduced positive cells of IL-1β, MMP13, and p-P65 on EP tissues in immumohistochemical staining (Figure 8).

## Discussion

Intervertebral disk degeneration is considered to be a major cause of LBP. Unfortunately, the etiological agent and progression of IVDD are still not well clarified (Scheele et al., 2012; Wang et al., 2012). Numerous studies have characterized disk degeneration as a multi-factorial disease caused by genetic and environmental factors (Chan et al., 2006; Manchikanti et al., 2009). IVDD manifests as loss of disk height, cell remodeling and apoptosis in the NP, disorganization of the AF, and fusion and ossification of EPs. In general, changes in the NP during IVDD are of utmost concern (Inoue and Espinoza Orias, 2011; Iatridis et al., 2013); however, changes in the EPs have not been studied as extensively; thus, this study examined how to protect the EPs during IVDD. The results of this study demonstrated that exogenous Mel protects EPCs from degeneration through decreasing inflammatory response and degradation of ECM induced by NF-κB pathway after binding to its specific receptors *in vitro* and exerts an alleviative effect on IVDD by protecting the EPs and its subchondral bones from damage *in vivo*.

The vertebral EPs cover the upper and lower surfaces of the IVD as homogeneous hyaline cartilage to ensure that the NP maintains hydrostatic pressure and to allow the transfer of nutrients, oxygen, metabolic products, and water (Antoniou et al., 1996). The IVD is the largest avascular tissue in the body; thus, a sufficient nutrient supply is necessary to avoid disk degeneration (Nachemson et al., 1970; Oegema, 1993). There is evidence that most of the nutrients transported from the surrounding capillaries are grown in the EPs (Holm et al., 1981). Therefore, compromising the integrity of the EPs could lead to a lack of nutrients and accumulation of metabolites (Rajasekaran et al., 2004; Cinotti et al., 2005). Likewise, the IVD will become unstable as cartilage is destroyed (Fontana et al., 2015). In the current study, rats with IVDD induced by needle puncture exhibited a loss of the NP, a disordered arrangement of AF, and concurrent fusion and ossification of the EPs. The vascular channels of the cartilage EP disappeared, resulting in fewer nutrients, eventually aggravating the IVDD. These results indicate that degeneration in the EPs is involved in the process of IVDD and, in turn, accelerate IVDD progression.

Mel is an endogenous hormone mainly secreted by the pineal gland and is regulated by the body’s circadian rhythms (Reiter, 1991). It was first identified in the late 1950s, and various studies have since confirmed that it plays a role in various functions, such as sleep propensity, thermoregulation, sexual maturation, antiproliferation, and immune response (Maestroni, 1993; Haimov et al., 1994; Pandi-Perumal et al., 2008). Mel functions by combining with Mel receptors, especially type 1a, which is the main one expressed in mammals (Brzezinski, 1997). Turgut et al. (2006b) proved that the decrease in Mel observed with aging was related to IVDD. Additionally, in a previous study, they found that an absence of Mel, caused by pinealectomy, accelerated IVDD in chicks, further confirming the putative role of Mel in the development of IVDD (Turgut et al., 2006a). In the present study, it was discovered that intraperitoneal injection of exogenous Mel plays a protective role in remedying IVDD in rats in a dose-dependent manner, which is consistent with previous studies.

Several investigators have examined the effects of Mel on bone physiology, on the basis of evidence for the secretion of parathyroid hormone and calcitonin influenced by the control of circadian rhythms *via* Mel (Hakanson and Bergstrom, 1981; Hakanson et al., 1987). Suzuki et al. (2008a, b) developed a synthetic Mel derivative for treating osteoporosis by inhibiting osteoclast activity. Experimental studies have shown that most functions carried out by Mel require binding to the Mel receptor (Cardinali et al., 2003; Witt-Enderby et al., 2006). In the present study, the expression of the Mel receptors 1a and 1b was detected in both the NP and EP in human, which consistent with Zheng et al. (Mao et al., 2017). Interestingly, due to the species differences between humans and rats, the two receptors were detected only in the EPs in rat, indicating that Mel may play an important role in alleviating IVDD by protecting the EPs in rats.

Furthermore, Mel inhibited the destruction and resorption of subchondral bones by downregulating RANKL expression and osteoclastogenesis, as numerous studies have reported the interaction of cartilages and subchondral bones in osteoarthritis (Funck-Brentano and Cohen-Solal, 2011; Karsdal et al., 2014). The destruction of the subchondral bone microstructure due to osteoporosis could further exacerbate experimental osteoarthritis (Bellido et al., 2010). Similarly, the cartilage EP was also found to be degenerated in the osteoporosis (Sun and Luo, 2019). The alteration of compression stiffness and strain distributions caused by subchondral bone disorders leads to further collapse of the EPs (Mattei, 2013). As a result, decreasing subchondral bone destruction might result in keeping the integrity of the EPs, and thus, nutrient supply was preserved.

On the other hand, our *in vitro* results found that Mel promoted the proliferation of EPCs at a concentration range of 1∼100 μM and, at the same time, protected the cell viability under the LPS condition through binding to its specific receptors. Several studies have demonstrated the proliferative effect of Mel on NP cells (NPCs), but very few of them paid attention to the influence of EPCs under Mel treatment (Ge et al., 2019). And as far as we know, this is the first time to demonstrate that Mel reveals the effect of promotion on proliferation and cell viability of rat EPCs following binding to its receptors. However, as Luzindole is a non-selective receptor inhibitor, the definite receptor that plays a role in it is still unclear; further studies are required to investigate it.

Moreover, Mel alleviated LPS-induced ECM degradation of rat EPCs through inhibiting the expression of inflammatory cytokines and MMPs activated by NF-κB pathway, and it was also blocked by the receptor inhibitor. NF-κB is a family of transcription factors that plays a central role in mediating cell response to damage, stress, and inflammation (Baker et al., 2011). Chen et al. (2020) reported that Mel alleviated IVDD by disrupting the IL-1β/NF-κB-NLRP3 inflammasome positive feedback loop in the NP, and similar to NPCs and EPCs, our results exhibited that the ECM degradation of rat EPCs caused by IL-1β and MMPs was also mediated by Mel through inhibiting NF-κB signaling. It is worth noting that the inflammatory process activated by NF-κB pathway has been found to be closely related to osteoclastogenesis and subsequent bone resorption (Ping et al., 2017). Therefore, combining the *in vivo* results above, we can suppose that the inhibitive effect of Mel on inflammatory response activated by NF-κB not only protects ECM degradation of the EPs from MMPs and ADAMTS-5 but also restrains the osteoclastogenesis in subchondral bones, which in turn prevents EP degeneration. Nevertheless, further studies are still needed to confirm it.

## Conclusion

In conclusion, the present study showed that Mel effectively alleviated IVDD caused by needle puncture in rats and protected ECM degradation of EPCs from MMPs and inflammatory cytokines activated by NF-κB both *in vitro* and *in vivo*. These effects were blocked by the non-selective receptor inhibitor, which indicates that Mel took effect in alleviating IVDD through binding to its specific receptors. IVDD is a global health problem for which novel therapies are needed. Mel, as an endogenous hormone secreted by the pineal gland, has been used as a health product in clinical practice and may be a new option for the treatment of IVDD.

## Data Availability Statement

The original contributions presented in the study are included in the article/supplementary material, further inquiries can be directed to the corresponding author/s.

## Ethics Statement

The animal study was reviewed and approved by the Ethics Committee of the First Affiliated Hospital of Soochow University (No. 201801A005). Human intervertebral disk tissues were obtained from the lumbar disks of scoliosis patients undergoing deformity correction surgery. The studies involving human participants were reviewed and approved by the Ethics Committee of the First Affiliated Hospital of Soochow University (No. 2019254). The patients/participants provided their written informed consent to participate in this study.

## Author Contributions

DG, HM, and HY: conceptualization. XW, YL, JD, XL, JL, LN, PZ, HZ, and FK: experiment. XW, YL, and JD: data analysis. HY, JL, LN, XL, PZ, HZ, and FK: methodology. DG, HM, and HY: original draft writing. DG, HM, and HY: manuscript review and editing. All the authors have read and agreed to publish the current version of the manuscript.

## Conflict of Interest

The authors declare that the research was conducted in the absence of any commercial or financial relationships that could be construed as a potential conflict of interest.
